# Towards robust data-driven reduced-order modelling for turbulent flows: application to vortex-induced vibrations

**DOI:** 10.1007/s00162-022-00609-y

**Published:** 2022-05-23

**Authors:** Yannick Schubert, Moritz Sieber, Kilian Oberleithner, Robert Martinuzzi

**Affiliations:** 1 Laboratory for Flow Instabilities and Dynamics, Technische Universität Berlin, Berlin, Germany; 2 Department of Mechanical and Manufacturing Engineering, University of Calgary, Calgary, AB Canada

**Keywords:** Reduced-order model, Vortex-induced vibration, Circular cylinder wakes, Spectral proper orthogonal decomposition, Nonlinear ODE system, Sparse systems

## Abstract

This work presents a robust method that minimises the impact of user-selected parameter on the identification of generic models to study the coherent dynamics in turbulent flows. The objective is to gain insight into the flow dynamics from a data-driven reduced order model (ROM) that is developed from measurement data of the respective flow. For an efficient separation of the coherent dynamics, spectral proper orthogonal decomposition (SPOD) is used, projecting the flow field onto a low-dimensional subspace, so that the dominating dynamics can be represented with a minimal number of modes. A function library is defined using polynomial combinations of the temporal modal coefficients to describe the flow dynamics with a system of nonlinear ordinary differential equations. The most important library functions are identified in a two-stage cross-validation procedure (conservative and restrictive sparsification) and combined in the final model. In the first stage, the process uses a simple approximation of the derivative to match the model with the data. This stage delivers a reduced set of possible library function candidates for the model. In the second, more complex stage, the model of the entire flow is integrated over a short time and compared with the progression of the measured data. This restrictive stage allows a robust identification of nonlinearities and modal interactions in the data and their representation in the model. The method is demonstrated using data from particle image velocimetry (PIV) measurements of a circular cylinder undergoing vortex-induced vibration (VIV) at $\mathrm{Re}=4000$. It delivers a reduced order model that reproduces the average dynamics of the flow and reveals the interaction of coexisting flow dynamics by the model structure.

## Introduction

In many engineering applications, the prediction and control of highly energetic coherent flow structures are important in designing, monitoring and optimising fluid systems. Most flows of practical interest are turbulent, and many strategies aim to gain insight into the flow dynamics by approximating only the coherent dynamics of the system with a reduced-order model (ROM). Over the last decades, refinements in experimental and numerical simulation methods have enabled access to very large amounts of high-fidelity data, making data-driven approaches an increasingly interesting choice for deriving ROMs [10]. However, the stochastic nature of turbulent flows poses two inherent challenges for data-driven approaches. First, the posterior system identification requires extracting deterministic dynamics, which are to be represented by low-order models, from direct observations of the stochastic flow process. Second, the calibration procedure must account for random perturbations of the flow that are extraneous to the candidate model and may lead to overfitting. This work presents a generic approach, which uses a two-stage cross-validation procedure for identifying a low-dimensional system to represent deterministic flow dynamics, to obtain a robust and accurate ROM. This is different form related investigations that use a statistical approach to separate the deterministic and stochastic dynamics in the data [17, 36]. The latter relies on the evaluation of probability density functions of the state variables to infer a stochastic state space model. The main benefit of the proposed cross-validation procedure over statistical approaches is that it requires significantly less data, especially for increasing dimensions of the model.

Many approaches to separate fluid dynamics efficiently are based on modal decomposition techniques. Generally, spatial flow structures are represented by modes and their temporal variations are captured by the corresponding modal coefficients. This allows a representation of the flow dynamics by a reduced set of modes and a simpler description of the dynamics through the temporal evolution of the modal coefficients [10, 24]. Prevalent examples are the decomposition into Fourier modes, dynamic mode decomposition (DMD) [31], proper orthogonal decomposition (POD) [1] and many variations and modified versions of these techniques [39]. Most commonly the modal base of low-order models is constructed from an energy-ranked POD of the flow data [40]. However, a common shortcoming of POD is the lack of a clear separation of individual dynamics into distinct modes, which makes it difficult to build efficient models. SPOD has been developed with the intent to distinguish different dynamics [35] and mitigate this shortcoming of classical POD. The SPOD seeks a short-time temporal coherence in addition to the spatial coherence that is imposed by the modal decomposition. The SPOD used here is based in the time domain and is different from the frequency-domain method with the same name described by Towne et al. [42]. Both SPOD variants share the inclusion of the short-time temporal correlation as detailed by Sieber [34]. However, the SPOD used in the current investigation is implemented completely in the time domain, whereas the other SPOD employs a Fourier transform of the temporal evolution, seeking correlations in the frequency domain. The time-domain SPOD provides a natural way to describe the time dependence of the flow through the expansion coefficients without the need for an ambiguous inverse transformation required in the frequency-domain SPOD [23]. These time-dependent expansion coefficients constitute the backbone for the data-driven reduced order model. In the following, SPOD refers to the time-domain version of SPOD.

In contrast to classical POD, SPOD requires the specification of the temporal correlation domain defined by a filter size (window function). The filter size can be selected based on the governing time scales of the flow, commonly the period time of a dominant oscillation of interest. This decomposition has proven to be a good choice in the following applications. SPOD has shown to be very beneficial for building ROMs based on deep neural networks [22]. It was used also to study sparse models of modal interactions in a cavity flow [28]. Furthermore, through SPOD it was possible to identify and explain the interaction of an acoustic and a hydrodynamics instability in a combustor from experimental data [38]. In a separate study of a swirl stabilised combustor, two coexisting dominant modes could be separated and attributed to coexisting global hydrodynamic instabilities [43]. Applications of SPOD enabled the identification of coherent structures in biofluid dynamics [7]. From the applications, it is clear that SPOD allows the coherent dynamics in turbulent flows to be partitioned into modes that have interpretable dynamics, mostly related to specific hydrodynamic phenomena. This is advantageous for the construction of ROMs, since the correct separation of coexisting phenomena is essential for the construction of efficient and interpretable ROMs.

Recently, machine learning approaches have been introduced in fluid dynamics such as sparse identification of nonlinear dynamics (SINDy) [5]. These use modal decomposition as an input to span a possible function space and sparse regression to identify relevant terms, with the objective of achieving a parsimonious model. This technique showed that efficient representations of canonical fluid dynamic problems can be derived with a sparse system of nonlinear, ordinary differential equations (nODE). However, without precise tuning, adaption and prior knowledge of the flow, the applicability to more complex problems and noisy measurement data has proven challenging. Alternative approaches which are gaining increasing interest are deep neural networks (DNN) [15]. These computational models are inspired by the neural structure of brains. The DNNs are composed of multiple layers through which input features are transformed to capture complex interactions [19]. In contrast to the SINDy algorithm, a trained neural network is used for the representation of the system dynamics [22]. Due to the complexity of these networks, the interpretation of physical relationships becomes more challenging and the extrapolation and robustness of the model need to be ensured. While DNNs show better performance for some flows [22], it comes at the expense of extensive training using high-fidelity data, higher computational efforts and the tacit risk of overfitting.

The data-driven identification of simplified equations from measurement or simulation data provides an efficient alternative to simulations based on Navier–Stokes equations. The present methodology is especially designed for an initial investigation of turbulent flows, as it does not require a priori knowledge of underlying models or dynamics. Further, it uses as few exogenous parameters as possible without resulting in trivial solutions. Projecting flow data onto a finite-dimensional subspace and using a reduced number of modes as a set of basis functions allows finding a formulation for the deterministic flow dynamics as a system of nODE. Then, with comparatively low computational effort, the implemented two-stage cross-validation (conservative and restrictive sparsification) results in a robust ROM. The ROM retains the capability of reproducing not only the flow statistics, but also provides insight of the physical interactions that can be deduced from the model structure and parameters. This approach is motivated by the potential benefits to engineering applications approximating real-time solutions, which could be used in, e. g. active flow control [14].

The proposed method is broadly comparable to the framework of the SINDy method. It similarly relies on a modal decomposition and the representation of nonlinear dynamics by an appropriate function library. The use of a polynomial library was motivated before by Noack’s xAMC method (now called xROM toolkit [33]), relying on the POD-based Galerkin projection of the Navier–Stokes equations [24]. The SINDy method augments xROM by introducing sparsification to the model. However, depending on the study, the way the sparsity of the system in enforced varies. Originally, a sequential thresholding procedure was proposed that does not balance the model sparsity against fidelity [5]. The resulting model sparsity directly depends on an arbitrarily selected iteration counter to prevent the algorithm to converge on the trivial limit of a stationary model. Further refinements of the SINDy approach suggest regularisation [20] that balance model complexity against fidelity similar to the LASSO method [29, 41]. This balance relies on regularisation parameters and further cause a convex optimisation problem that is harder to solve. Furthermore, the user-selection of the regularisation parameters directly controls the model complexity and needs to be varied and adjusted from case to case [9]. The method proposed in this work tries to avoid the direct dependency of the model complexity on user selected parameters. This is achieved by a sequential evaluation of parameters in a brute-force manner and relies on cross-validation to evaluate the model fidelity. In summary, the method proposed here replaces the regularised minimisation problem used in the SINDy method. Note that the utilised SPOD-based ROM is related to the HAVOK approach [4] and can therefore be considered as convolutional coordinates [12], as explained in more detail in [34].

The method is demonstrated for two-dimensional PIV data in the wake of a circular cylinder at $\mathrm{Re}=4000$ undergoing VIV. Exposing an elastically mounted cylinder to a fluid flow can induce a cylinder vibration due to vortex shedding. The vortex shedding causes oscillating pressure fluctuations which apply forces onto the cylinder inducing a motion. This modifies the dynamics of the vortices, leading to a nonlinear fluid-structure interaction. The response (oscillation amplitude) of the cylinder depends on the oncoming stream velocity, $U_\infty$, and the natural frequency of the cylinder, $f_n$, and is classified through four regimes characterised through the reduced velocity $U^*=U_{\infty }/Df_n$, with *D* the cylinder diameter. These regimes are the: (i) initial excitation regime; (ii) "upper branch" with a very high amplitude response; (iii) "lower branch" with a moderate amplitude response; and (iv) a desynchronisation regime [13]. Within these regions, certain wake pattern can be observed [44]. When the normalised velocity is gradually increased within the "upper" and "lower branch", a "lock-in" phenomenon can be observed, where the vortex shedding frequency synchronises with the cylinder oscillation. Further increase in the velocity leads to a desynchronisation of the vortex shedding and cylinder motion. VIV problems have been intensively studied over the last decades, as it entails several engineering challenges and covers numerous of disciplines. A good review of applied methods is provided in [30].

This work is organised as follows: Sect. 2 provides a detailed description of the methodology, introducing the modal decomposition technique (SPOD) and proposing a quantifying method to identify coupled mode pairs with a DMD (Sect. 2.1). The new algorithms of the two-staged sparsification procedure (conservative and restrictive sparsification) are presented in Sect. 2.2. In Sect. 3, the method is demonstrated for PIV data of circular cylinder undergoing VIV in the desynchronisation regime. The results and findings are outlined in Sect. 4.

## Methodology

**Fig. 1:**
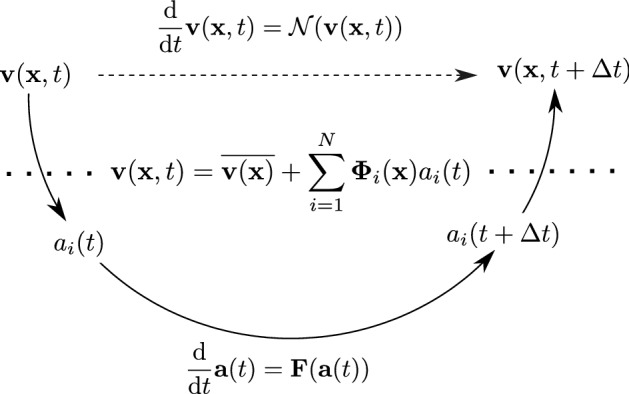
Conceptual schematic for deriving the temporal evolution of the flow field: the dashed arrow is the direct approach solving Navier–Stokes equations (theoretical solution), curved arrows indicate the indirect approach via a modal decomposition (applied solution); the dotted line indicates the transition between physical (above) and modal space (below)

The aim of this work is to identify a simplified system for efficiently representing the dominant flow dynamics. As schematically shown in Fig. 1, instead of directly describing the temporal evolution of the flow field $\mathbf {v}(\mathbf {x},t)$ with the Navier–Stokes equations ($\mathcal {N}$), the basis of the problem is changed, using a modal decomposition to approximate the instantaneous velocity field with only a few basis functions. Then, the evolution of the temporal coefficients $a_i(t)$ is represented with a nonlinear operator $\mathbf {F}$. In a final step, the approximated field is projected back to the canonical basis.

**Fig. 2:**
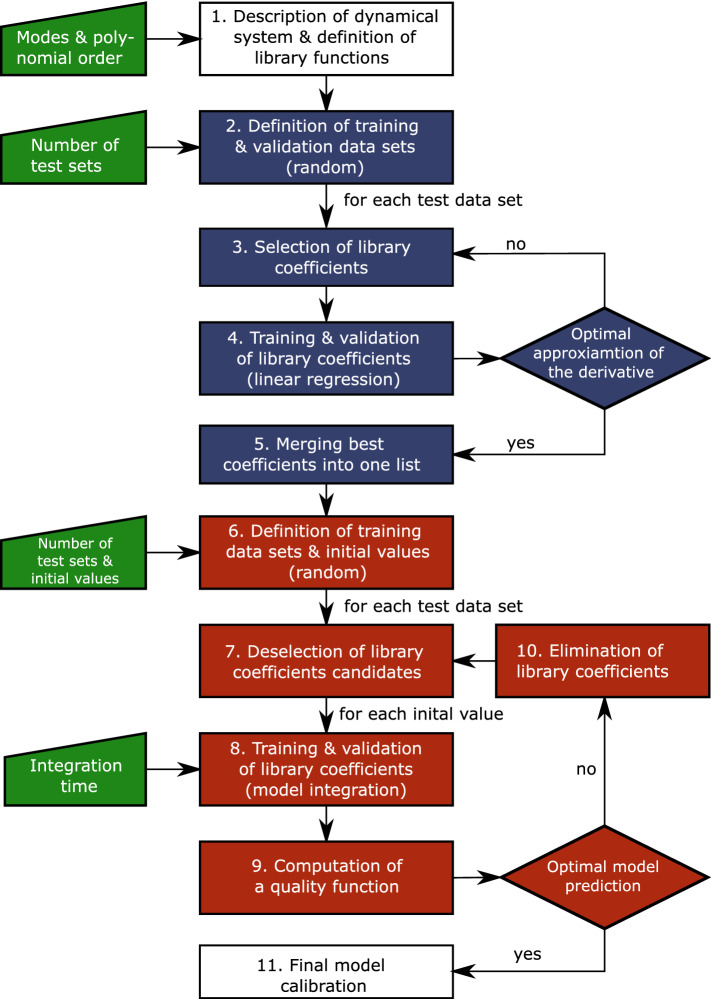
Process flow chart grouped conceptually by operations: conservative/preliminary sparsification (yellow), restrictive sparsification (purple), input parameters (green)

The general process can be divided into two main steps: first, reducing the dimensionality of the input data by a modal decomposition and, second, building robust ROM from data to approximate the nonlinear operator $\mathbf {F}$ (sparsification procedure). A flow chart summarizing the steps of the two sparsification algorithms is shown in Fig. 2.

### Flow field decomposition and mode coupling

In this section, the essential steps of the data decomposition (SPOD) and a method to find coupled modes are presented following Sieber et al. [35]. Data, either measured (e. g. PIV) or computed (CFD), are recorded as snapshots (*M* spatial points over *N* time steps).

For PIV and CFD data, usually $M\ge N$ and the initial decomposition steps are more suitably carried out with the snapshot POD [37]. The POD projects the data onto an orthogonal base that is optimal with respect to the representation of kinetic energy of fluctuations, so that the most energetic structures can be represented by a minimal number of basis functions. As a first step, the velocity field $\mathbf {v}(\mathbf {x},t)$ is decomposed into a mean velocity field $\overline{\mathbf {v}(\mathbf {x})}$ and fluctuations $\mathbf {v}'(\mathbf {x},t)$. For the VIV data, the flow shows a spatial symmetry. Following Holmes et al. [10], this property is exploited and the decomposition is performed for a symmetrised flow field using the procedure described in “Appendix A”. By reinforcing natural symmetries of the modes, the convergence rates are increased with respect to the number of employed snapshots [3]. Note that this procedure also optimises the spatial convergence of the later applied filter operation of the SPOD.

Thus, as shown in Fig. 1, a decomposition of $\mathbf {v}(\mathbf {x},t)$ in the form

1 $$\begin{aligned} \mathbf {v}(\mathbf {x},t)&=\overline{\mathbf {v}(\mathbf {x})}+\sum _{i=1}^{N}{{\varvec{\Phi }}_{i}(\mathbf {x})b_{i}(t)}, \end{aligned}$$

is sought, where ${\varvec{\Phi }}_{i}(\mathbf {x})$ are the spatial modes and $b_{i}(t)$ the corresponding temporal coefficients. The modal basis is constructed from correlated observations of fluctuations in the flow. Therefore, the correlation between individual snapshots is computed using the $L^2$ inner product:

2 $$\begin{aligned} \langle \mathbf {u}(\mathbf {x},t_i), \mathbf {v}(\mathbf {x},t_j)\rangle = \int _{\mathrm {\Gamma }} \mathbf {u}(\mathbf {x},t_i)\mathbf {v}^\mathrm {T}(\mathbf {x},t_j)\mathrm {dS}, \end{aligned}$$

where $\mathrm {\Gamma }$ specifies the spatial region (PIV window) of integration. The elements of the covariance matrix $\mathbf {C}$ represent the covariance over all snapshots and are given by

3 $$\begin{aligned} C_{ij}=\frac{1}{N}\langle \mathbf {v}'(\mathbf {x},t_i), \mathbf {v}'(\mathbf {x},t_j)\rangle . \end{aligned}$$

At this point, the only additional step of SPOD is applied. Instead of directly deriving the temporal coefficients with an eigendecomposition of $\mathbf {C}$, an additional filter operation is applied to the covariance matrix, which requires the selection of a filter. The filter is specified by the size of a discrete filter in terms of sampling points $N_\mathrm {f}$, which is equivalent to a short time span $N_\mathrm {f} \varDelta t$ given the sampling interval of the signal $\varDelta t$. The elements of the resulting filter covariance matrix $W_{ij}$ are given by

4 $$\begin{aligned} W_{ij}&=\sum _{k=-N_\mathrm {f}}^{N_\mathrm {f}}g_{k}C_{i+k,j+k}. \end{aligned}$$

Here, a Gaussian filter is applied, where the vector elements $g_k$ are:

5 $$\begin{aligned} g_{k}=e^{-(2.285k/N_\mathrm {f})^2}. \end{aligned}$$

There are three options to deal with boundary points: (i) Periodic boundary conditions are imposed, if the periodic property applies to the investigated flow. (ii) Invalid boundary points are removed. If the filter operation relies on samples outside the acquired time frame, the analysis window is reduced (the option applied to the VIV data). This requires much smaller filter sizes than the number of snapshots to retain sufficient data after the truncation. (iii) Adding zeros to the end of the signal (zero padding). The filter operation allows a continuous transition of the decomposition between the POD and the discrete Fourier transform (DFT). Depending on the filter size, the temporal coefficients of the SPOD modes are better behaved than POD coefficients, i. e. the time evolution is smoother and with reduced spectral bandwidth. Thus, these coefficients are more suitable for deriving a ROM, especially for turbulent flows. The filter size is the only additional parameter compared to POD and should be chosen with respect to a characteristic time scale of the flow. For the VIV measurement, the selected scale corresponds to two periods of the cylinder oscillation. With the eigendecomposition of the filtered covariance matrix, the temporal coefficients $b_i(t)$ and the corresponding eigenvalues $\mu _i$ (representing twice the average kinetic energy of each mode) can be obtained

6 $$\begin{aligned} \sum _{k=1}^{N}W_{jk}b_i(t_k)=\mu _i b_i(t_j),\quad \mu _1\ge \mu _2 \ge \dots \ge \mu _N. \end{aligned}$$

Since $\mathbf {W}$ is a real symmetric positive-definite matrix, its eigenvectors $b_i(t)$ are orthogonal and scaled with the mode energy

7 $$\begin{aligned} \frac{1}{N}\sum \limits _{k=1}^Nb_i(t_k)b_i(t_k)=\mu _i. \end{aligned}$$

From the projection of the snapshots onto the temporal coefficients, the spatial modes can be derived

8 $$\begin{aligned} {\varvec{\Phi }}_{i}(\mathbf {x})=\frac{1}{N\mu _i}\sum _{j=1}^{N}b_i(t_j)\mathbf {v}'(\mathbf {x},t_j). \end{aligned}$$

The identification of mode pairs, typically having a similar amount of kinetic energy, but shifted in phase, is essential to represent the dynamics of coherent structures. Most approaches for identification involve an inspection of the spatial modes shapes and Lissajous figures, which becomes more difficult for complex dynamics. Sieber et al. [35] introduce an unbiased method to quantify coupled dynamics of modes, using DMD. The idea is to find temporal coefficients with the same spectral content, considering a $\pi /2$ phase lag to calculate a spectral proximity measure. Therefore, it is assumed that the temporal evolution of the mode coefficients is captured by a linear operator $\mathbf {T}$:

9 $$\begin{aligned} \mathbf {b}(t+\varDelta t)=\mathbf {T}\mathbf {b}(t). \end{aligned}$$

With two snapshot matrices $\mathbf {V}_1$ and $\mathbf {V}_2$ defined as follows

10 $$\begin{aligned} \mathbf {V}_1&:=\left[ \mathbf {{b}}(0) \quad \mathbf {b}(\varDelta t) \quad \dots \quad \mathbf {b}((N-2)\varDelta t)\right] , \end{aligned}$$

11 $$\begin{aligned} \mathbf {V}_2&:=\left[ \mathbf {b}(\varDelta t) \quad \mathbf {b}(2\varDelta t) \quad \dots \quad \mathbf {b}((N-1)\varDelta t)\right] , \end{aligned}$$

the operator $\mathbf {T}$ can be described by

12 $$\begin{aligned} \mathbf {V}_2&= \mathbf {T} \mathbf {V}_1, \end{aligned}$$

13 $$\begin{aligned} \mathbf {T}&= \mathbf {V}_2 \mathbf {V}_1^{+}, \end{aligned}$$

where $\mathbf {V}_1^{+}$ is the Moore–Penrose pseudo-inverse of $\mathbf {V}_1$. Note, that the explicit computation of $\mathbf {V}_1^{+}$ can be problematic for ill-condition snapshot matrices and Eq. (13) is only used for illustration. The problem can also be described and solved by a least-square regression. Hence, the latter option is used in the implementation of the procedure using the MATLAB backslash operator. The DMD modes can then be derived by an eigendecomposition of the operator $\mathbf {T}$:

14 $$\begin{aligned} \mathbf {T}\mathbf {h}_i&=\nu _i\mathbf {{h}}_i. \end{aligned}$$

The modal representation of $\mathbf {b}(t)$ can be written as

15 $$\begin{aligned} b_{j}(t)=\sum _{i=1}^{N_\mathrm {c}}{h_{ij}\mathrm {e}^{ \frac{\ln {(\nu _i})}{\varDelta t}t}}, \end{aligned}$$

where $h_{ij}$ is the spectral content of the single mode coefficient $b_{j}$ with respect to the eigenvalue $\nu _i$. Note, that this notation is only exact for $N_\mathrm {c}=N$; however, for the described process $N_\mathrm {c}<N$. To mitigate measurement noise in the identification procedure, only modes with an acceptable signal-to-noise ratio should be considered for the calculation of $\mathbf {T}$. Modes with very low energy are associated with a low signal-to-noise ratio. To remove these modes, the number of retained modes (sorted by energy in descending order) is truncated after $N_\mathrm {c}$ modes. This number is calculated based on the energy resolved by the SPOD:

16 $$\begin{aligned} E(N_\mathrm {c})&=\frac{\sum \nolimits ^{N_\mathrm {c}}_{k=1}\mu _k}{\sum \nolimits ^{N}_{k=1}\mu _k}. \end{aligned}$$

For the VIV measurement, the modes are truncated around $E(N_\mathrm {c})=0.95$. The amplification rates $\sigma _i$ and frequencies $\omega _i$ of the operator $\mathbf {T}$ are related to eigenvalue $\nu _i$ as follows:

17 $$\begin{aligned} \frac{\ln {(\nu _i)}}{\varDelta t}=\sigma _i+ \mathrm {i}\omega _i, \end{aligned}$$

where $\text {i}:=\sqrt{-1}$. The measure of the proximity for ranking the spectral similarity of temporal coefficients $\mathbf {b}(t)$ is given by

18 $$\begin{aligned} H_{ij}=\Im \left\{ \sum _{k=1}^{N_\mathrm {c}}{h_{ki}h^*_{kj}\,\text {sgn}({\text {Im}}(\nu _k))}\right\} =-H_{ji}. \end{aligned}$$

The elements of the skew-symmetric matrix $\mathbf {H}$ are sorted by decreasing magnitude. The first SPOD mode corresponds to the largest element $H_{mn}$ identifying the coupled modes *m* and *n*. The next lower magnitude of $\mathbf {H}$, excluding the row and column of the first element, defines the second coupled SPOD modes, and the process is repeated until all modes are paired. The coupled modes $b_m$ and $b_n$ form the real and imaginary parts of a complex SPOD mode $a_i(t)$ with an amplitude $A_i(t)$ and a phase $\varphi _i(t)$

19 $$\begin{aligned} a_i(t)&=b_m(t)+ \mathrm {i}b_n(t)=A_i(t)\mathrm {e}^{\mathrm {i}\varphi _i(t)} \text {, for fixed index pairs } (m,n). \end{aligned}$$

The subscript *i* corresponds to the sorting order. The spatial modes are paired analogously:

20 $$\begin{aligned} \varPsi _i(t)&=\varPhi _m(t)- \mathrm {i}\varPhi _n(t) \text {, for fixed index pairs } (m,n). \end{aligned}$$

The entire time-dependence is captured by the state vector $\mathbf {a}(t)$. Using the complex definition of the modes, the reconstruction equivalent to Eq. (1) now reads:

21 $$\begin{aligned} \mathbf {v}(\mathbf {x},t)&=\overline{\mathbf {v}(\mathbf {x})}+\sum _{i=1}^{N}\Re {({{\varvec{\Psi }}_{i}(\mathbf {x})a_{i}(t))}}, \end{aligned}$$

Thus, the temporal evolution of the flow can be represented by deriving a ROM for the nonlinear operator $\mathbf {F}$ to describe the deterministic dynamics of the flow:

22 $$\begin{aligned} \frac{{\mathrm {d}}}{{\mathrm {d}} t}\mathbf {a}(t)&=\mathbf {F}(\mathbf {a}(t)). \end{aligned}$$

### Sparsification

While the decomposition of the velocity fluctuations into SPOD modes in Eq. (1) is exact (when all modes are used), it is desired to select a function basis using the least number of modes $N_\mathrm {m}$, while describing the dominant coherent dynamics sufficiently well.

#### Mode selection

The mode selection depends on the flow configuration, and the following points need consideration. The SPOD procedure already ranks modes in two ways. The kinetic energy ranking Eq. (6) considers the larger energetic contributions, and the harmonic correlation ranking Eq. (18) provides insight into the possible dynamic significance of the lower energy interactions. The highest ranked modes of both sortings should be selected. Note, that the highest ranked modes in both cases do not have to differ remarkably. The final model prediction can also provide insights, if sufficient modes have been selected to cover the most important interactions. For the VIV data, the first three SPOD modes with the highest energy are also those with the highest harmonic correlation. These represent the vortex shedding synchronised with the cylinder oscillation, the natural vortex shedding, and their dominating nonlinear interaction.

#### Computation of the derivative

To describe the deterministic dynamics, the derivative of the selected modes provides the basis for the two-stage sparsification. In order to accurately estimate the numerical derivative from data, while avoiding error amplification due to measurement noise, a sixth-order compact scheme [18] was chosen. For each temporal coefficient, the unknown derivative $\dot{\mathbf {a}}(t)$ can be obtained by solving the tridiagonal linear system:

23 $$\begin{aligned} \delta _1\dot{\mathbf {a}}_{j-1}+\dot{\mathbf {a}}_{j}+\delta _1\dot{\mathbf {a}}_{j+1}=\delta _2\frac{{\mathbf {a}}_{j+2}-{\mathbf {a}}_{j+2}}{\varDelta t}+\delta _3\frac{{\mathbf {a}}_{j+1}-{\mathbf {a}}_{j+1}}{\varDelta t}. \end{aligned}$$

Here, $\varDelta t$ is the time step between two snapshots and the numerical coefficients for all inner points $j\in {3,..,N-2}$ of the derivative stencil are:

24 $$\begin{aligned} \delta _1=1/3, \delta _2=14/18, \delta _3=1/36. \end{aligned}$$

The definition at the boundary is shown in “Appendix C”. Both the state vector and its derivative can be arranged in two matrices:

25 $$\begin{aligned} \mathbf {A}&= \begin{bmatrix} {\mathbf {a}}^T (t_1) \\ {\mathbf {a}}^T (t_2) \\ \vdots \\ {\mathbf {a}} ^T (t_N) \\ \end{bmatrix} = \begin{bmatrix} {a}_1(t_1) &{}{a}_2(t_1) &{} \dots &{}{a}_{N_\mathrm {m}}(t_{1}) \\ {a}_1(t_2) &{} {a}_2(t_2) &{} \dots &{}{a}_{N_\mathrm {m}}(t_{2}) \\ \vdots &{} \vdots &{} \ddots &{}\vdots \\ {a}_1(t_{N}) &{}{a}_2(t_{N}) &{} \dots &{}{a}_{N_\mathrm {m}}(t_{N}) \\ \end{bmatrix} \end{aligned}$$

26 $$\begin{aligned} \dot{\mathbf {A}}&= \begin{bmatrix} \dot{\mathbf {a}}^T (t_1) \\ \dot{\mathbf {a}}^T (t_2) \\ \vdots \\ \dot{\mathbf {a}} ^T (t_N) \\ \end{bmatrix} = \begin{bmatrix} \dot{a}_1(t_1) &{} \dot{a}_2(t_1) &{} \dots &{}\dot{a}_{N_\mathrm {m}}(t_{1}) \\ \dot{a}_1(t_2) &{} \dot{a}_2(t_2) &{} \dots &{}\dot{a}_{N_\mathrm {m}}(t_{2}) \\ \vdots &{} \vdots &{} \ddots &{}\vdots \\ \dot{a}_1(t_{N}) &{} \dot{a}_2(t_{N}) &{} \dots &{}\dot{a}_{N_\mathrm {m}}(t_{N}) \\ \end{bmatrix}. \end{aligned}$$

#### Function library

It is assumed that the nonlinear operator in Eq. (22) can be described by a linear combination of a set of library functions, which are polynomial functions of the modal coefficients. For the present investigation of VIV data, polynomial combination of the coupled mode coefficients of $\mathbf {A}$ and its complex conjugate $\overline{\mathbf {A}}$ up to the cubic order are used to build the library ${\varvec{\Theta }}(\mathbf {A})$, which corresponds to the highest order of terms that would arise from a POD-based Galerkin projection of the Navier–Stokes equations [24]:

27 $$\begin{aligned} {\varvec{\Theta }}(\mathbf {A}) = \begin{bmatrix} \mid &{} \mid &{} \mid &{} \mid &{} \mid &{} \mid &{} \mid \\ 1 &{} \mathbf {A}&{} \overline{\mathbf {A}} &{} \mathbf {A}^{P_2} &{} \overline{\mathbf {A}}^{P_2} &{} (\mathbf {A}\overline{\mathbf {A}})^{P_2}&{} \mathbf {A}^{P_3}&{} \dots \\ \mid &{} \mid &{} \mid &{} \mid &{} \mid &{} \mid &{} \mid \\ \end{bmatrix}. \end{aligned}$$

Here, $\mathbf {A}^{P_2}$, $\mathbf {A}^{P_3}$, etc., denote higher order polynomials, where $\mathbf {A}^{P_2}$ denotes quadratic nonlinearities of the state vector $\mathbf {a}(t)$ and $(\mathbf {A}\overline{\mathbf {A}}^{P_2})$ denotes quadratic nonlinearities of the state vector $\mathbf {a}(t)$ and its complex conjugated $\mathbf {a}^*(t)$:

28 $$\begin{aligned} \mathbf {A}^{P_2}&= \begin{bmatrix} {a}_1(t_1)^2 &{} {a}_1(t_1){a}_2(t_1) &{} \dots &{} {a}_2(t_1)^2 &{} \dots &{} {a}_{N_\mathrm {m}}^2(t_{1}) \\ {a}_1(t_2)^2 &{} {a}_1(t_2){a}_2(t_2) &{} \dots &{} {a}_2(t_2)^2 &{} \dots &{} {a}_{N_\mathrm {m}}^2(t_{2}) \\ \vdots &{} \vdots &{} \ddots &{} \vdots &{} \ddots &{}\vdots \\ {a}_1(t_{N})^2 &{} {a}_1(t_{N}){a}_2(t_{N}) &{} \dots &{} {a}_2(t_{N})^2 &{} \dots &{} {a}_{N_\mathrm {m}}^2(t_{{N}}) \\ \\ \end{bmatrix} \end{aligned}$$

29 $$\begin{aligned} (\mathbf {A}\overline{\mathbf {A}})^{P_2}&= \begin{bmatrix} {a}_1(t_1){a}^*_1(t_1) &{} {a}_1(t_1){a}^*_2(t_1) &{} \dots &{} {a}_1(t_1){a}^*_{N_\mathrm {m}}(t_1) &{} \dots &{} {a}_{N_\mathrm {m}}(t_{1}){a}^*_{N_\mathrm {m}}(t_{1}) \\ {a}_1(t_2){a}^*_1(t_2) &{} {a}_1(t_2){a}^*_2(t_2) &{} \dots &{} {a}_1(t_2){a}^*_{N_\mathrm {m}}(t_2) &{} \dots &{} {a}_{N_\mathrm {m}}(t_{1}){a}^*_{N_\mathrm {m}}(t_{1}) \\ \vdots &{} \vdots &{} \ddots &{} \vdots &{} \ddots &{}\vdots \\ {a}_1(t_{N}){a}^*_1(t_{N}) &{} {a}_1(t_{N}){a}^*_2(t_{N}) &{} \dots &{} {a}_1(t_{N}){a}^*_{N_\mathrm {m}}(t_{N}) &{} \dots &{} {a}_{N_\mathrm {m}}(t_{N}){a}^*_{N_\mathrm {m}}(t_{N}) \\ \end{bmatrix}. \end{aligned}$$

Generally, the library may be composed of all possible forms of nonlinear functions and polynomial combinations of the state vector to account for nonlinear dynamics. In the next step, linear-dependent library functions are removed from the library, using a *Q*-*R*-decomposition.

#### Sparsification procedure

Assuming that only a few of the library functions are required for the representation of $\mathbf {F}$, a sparse regression problem can be formulated. The idea is to determine the important library coefficients, i. e. finding the sparse vectors of library coefficients ${\varvec{\Xi }}=[{\varvec{\xi }}_1 \, {\varvec{\xi }}_2 \, \dots \, {\varvec{\xi }}_{N_\mathrm {m}}]$ for each mode. Hence, Equation ( 22) can be formulated as

30 $$\begin{aligned} \dot{\mathbf {A}}={\varvec{\Theta }}(\mathbf {A}){\varvec{\Xi }}. \end{aligned}$$

Nonzero entries (active coefficients) of ${\varvec{\xi }}_k$ define which of the library functions are contributing to compute mode coefficient $a_k(t)$. Initially, all coefficients are considered possible candidates.

**Fig. 3:**
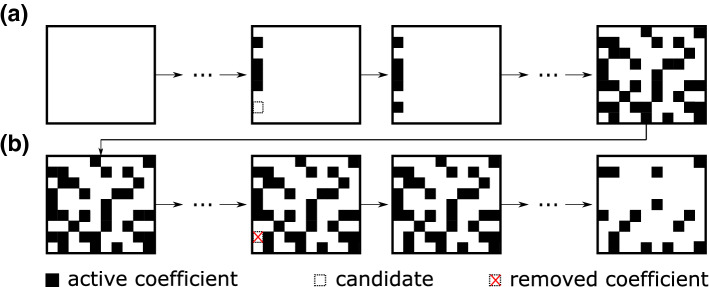
Conceptualisation of the library coefficient matrix in a) conservative sparsification b) restrictive sparsification

The sparsification procedure seeks to identify the important, i. e. statistically significant library coefficients and thereby reduces the model complexity. This avoids the risk of overfitting and increases the robustness of the ROM, while maintaining its accuracy. The procedure consists of two cross-validation stages, which allow to evaluate the statistical importance of individual coefficients and minimise computational costs. A simplified conceptual schematic of the process for both stages is depicted in Fig. 3.

The first stage (conservative sparsification) focuses on the approximation of the derivative and the second stage (restrictive sparsification) on the model prediction. In both stages, the selected library coefficients are trained with a subset of the data based on the optimal approximation of the derivative and validated against the remaining data. For this, the measurement data are split into continuous data segments of length $N_\mathrm {s}$. Here, the length is based on the SPOD filtering operation ($N_\mathrm {s}=N_\mathrm {f}$). Then, these segments are $N_{\mathrm {train}}$ times randomly assigned to training $\mathbf {a}^\mathrm {T}\left( \mathbf {t}^{\mathrm {train}}\right)$ and validation $\mathbf {a}^\mathrm {T}\left( \mathbf {t}^{\mathrm {val}}\right)$ data. Based on exploratory trials using different distribution ratios, results using 20% data segments for training and 80% for validation yielded consistently robust models. The ratio should be chosen with respect to the amount of available data maximising the validation data while keeping enough data for training to reduce the risk of overfitting. For the VIV data, $N_{\mathrm {train}}=100$ showed good convergence for both stages with respect to the final selection of the library coefficient.

#### Conservative sparsification

The calculation procedure for the conservative sparsification is presented in Algorithm 1. The principle of this cross-validation step is a bottom-up approach of a greedy algorithm to approximate the derivative for each mode, i. e. finding the optimal choice of a library coefficient in each iteration to improve the approximation of the derivative. Initially, all library coefficients are set passive, i. e. set to zero and excluded from training, and the individual coefficients are gradually activated, trained and evaluated. The training of the library coefficients for the *k*th individual mode for the *i*th training data set is computed as follows:

31 $$\begin{aligned} {\varvec{\xi }}^\mathrm {min}_{k}&=\mathop {\mathrm{argmin}}\limits _{{{\varvec{\xi }}}}\left\| \dot{a}^\mathrm {T}_{k}\left( \mathbf {t}^{\mathrm {train}}\right) -{\varvec{\Theta }}\left( a^\mathrm {T}_{k}\left( \mathbf {t}^{\mathrm {train}}\right) \right) {{\varvec{\xi }}}_{k}\circ \mathbf {d}^i_k\right\| \end{aligned}$$

32 $$\begin{aligned} {\varvec{\xi }}^\mathrm {train}_{k}&={\varvec{\xi }}_{k}^{\mathrm {min}}\circ \mathbf {d}^i_k. \end{aligned}$$

Here, the operator $\circ$ stands for an elementwise product and $\mathbf {d}^i_k$ is a Boolean vector controlling which coefficients are active and passive. Note, that this notation is chosen to indicate dependencies of the variables and to be in line with Algorithm 1, but the equations are correct even without the index for the training set variation. With the trained coefficients, the approximation residual $e_{jk}$ for the *j*th library function of the *k*th mode of the derivative can be derived:

33 $$\begin{aligned} e_{jk}=\left\| \dot{a}^\mathrm {T}_{k}\left( \mathbf {t}^{\mathrm {val}}\right) -{\varvec{\Theta }}\left( a^\mathrm {T}_{k}\left( \mathbf {t}^{\mathrm {val}}\right) \right) {\varvec{\xi }}_{k}^{\mathrm {min}} \right\| ^2. \end{aligned}$$

When all passive coefficients are individually tested, the library coefficient that shows the highest improvement of the residual in an iteration is kept (permanently) activated. This procedure is repeated until further activation of coefficients no longer result in improvements with respect to the residuals. This results in a set of possible candidates of library coefficients for each random training and validation data set, which are merged into a final list of possible candidates.

#### Restrictive sparsification

The calculation procedure of the restrictive sparsification is given in Algorithm 2. This procedure is a top-down approach, that starts with the candidate list of the conservative sparsification. In contrast to the first stage, the idea is to optimise the coefficient selection based on the model prediction on a system level and not for each mode individually. In an iterative procedure, models are built excluding one of the library coefficients candidates, trained and then, a short time integration is performed. As a reference, an additional integration with all candidates included is computed. The training of the library coefficients follows the same procedure as in the conservative sparsification algorithm, but for the entire system simultaneously:

34 $$\begin{aligned} {\varvec{\Xi }}^{\mathrm {min}}&=\mathop {\mathrm{argmin}}\limits _{{\varvec{\Xi }}}\left\| \dot{\mathbf {a}}^\mathrm {T}\left( \mathbf {t}^{\mathrm {train}}\right) -{\varvec{\Theta }}\left( \mathbf {a}^\mathrm {T}\left( \mathbf {t}^{\mathrm {train}}\right) \right) {\varvec{\Xi }}\circ \mathbf {D}\right\| \end{aligned}$$

35 $$\begin{aligned} {\varvec{\Xi }}^{\mathrm {train}}&={\varvec{\Xi }}^{\mathrm {min}}\circ \mathbf {D} , \end{aligned}$$

where $\mathbf {D}$ is a Boolean matrix that contains the information of currently active and passive library coefficients. Data points at the beginning of every non-training segment are used as initial values. This ensures an equal distribution and weighting of measurement data used as initial values, i. e. avoiding initial values taken from consecutive data points. As the equal-length segmentation is used on a periodic flow, the start of the segment is randomly shifted within the length of one oscillation period to prevent an over-weighting of a specific phase. To determine the deviation of the model prediction from the true state, the following initial value problem is $N_\mathrm {p}$ times solved (for the *n*th initial value $t^0$) and the residual is computed:

36 $$\begin{aligned} r_{n}= \int \limits _{t^{0,n}}^{t^{0,n}+N_\mathrm {t}\varDelta t}\left\| \mathbf {a}(t) - \mathbf {a}(t^{0,n}) -\int \limits _{t^{0,n}}^{t}F(\mathbf {a}(s))\mathrm {d}s \right\| ^2\mathrm {d}t. \end{aligned}$$

The number of simulated time steps $N_\mathrm {t}$ is based on two periods of the cylinder oscillation. A solver based on an explicit Runge–Kutta of fourth- and fifth-order (Dormand–Prince method) is used to integrate the model. If the integration is aborted (unstable model), the residual is penalised with ${r}_{0k}$. Simulations showed that this mainly occurs when the most important coefficients are excluded. With difference of the residuals (active and passive coefficient), a quality measure $\mathbf {Q}$ for each coefficient candidate can be computed (see Algorithm 2). The quality measure allows to determine, if a coefficient is statistically significant. Hence, all coefficients that are not statistically significant (negative values of $\mathbf {Q}$) are removed. With the new list (sparse matrix ${\mathbf {D}}$) of candidates, the procedure is repeated, until the list of candidates remains unchanged. In a final step, the resulting library coefficients (active coefficients in matrix ${\varvec{\Xi }}^{\mathrm {final}}$) are trained using the entire measurement data as training data. Then, with a simulation with resulting ROM of the temporal coefficients $\mathbf {a}^{\mathrm {sim,T}}(\mathbf {t})$ , the flow field can be reconstructed.
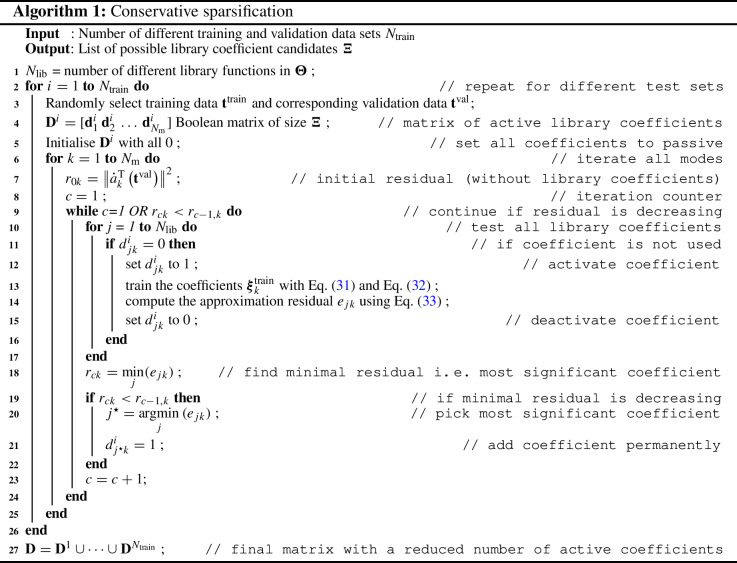

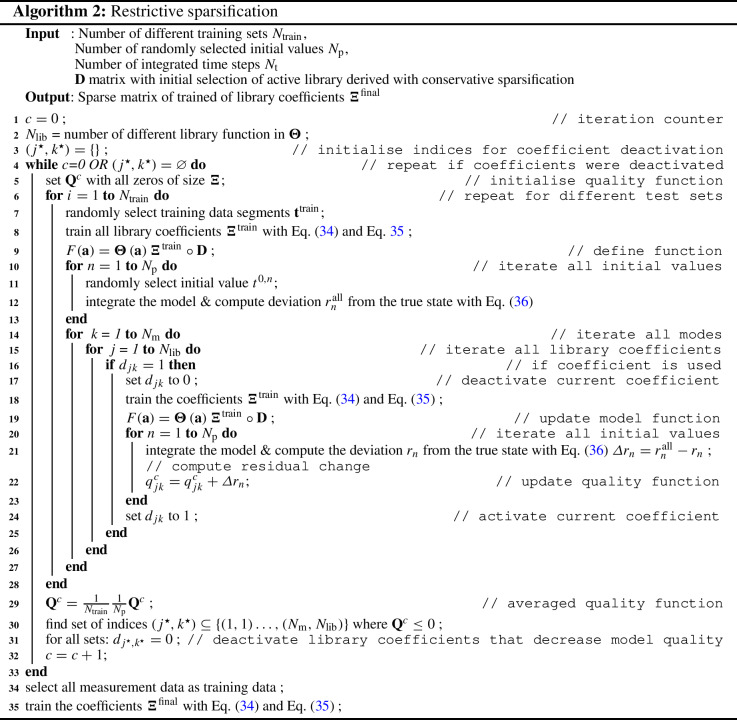


## Application to experimental data

In this section, the presented method is applied to experimental data of the VIV of a circular cylinder. The datasets analysed during the current study are available from the authors on reasonable request.

### Experimental setup

The experiments were carried out at the University of Calgary in a free-surface water channel facility. Details of the experimental setup can be found in “Appendix D” and Riches et al. [26]. This work focuses on the desynchronisation regime, as it involves complex dynamical interactions of both, the motion induced by the cylinder oscillation and the vortex shedding. In this regime, the spectral signature of the velocity fluctuations and the independently measured lift force show peaks at both the shedding frequency $f_\mathrm {sh}$ and cylinder vibration frequency, $f_\mathrm {c}$ allowing a good demonstration of the method. The experiments were conducted for a reduced velocity of $U^*=8.75$. The system has one degree of freedom with the cylinder moving transversely to the flow.

**Fig. 4:**
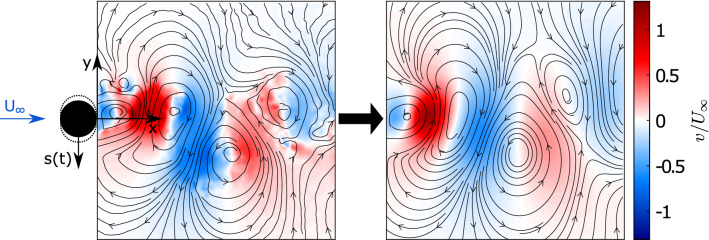
Basic representation of the VIV system: circular cylinder (black circle) with its maximal deflection (dashed lines), snapshot of the PIV window with the displayed *v*-component of the velocity field (blue and red) and streamlines (black) on the left and the reconstructed snapshot of the velocity field using the three most energetic SPOD modes on the right

**Fig. 5:**
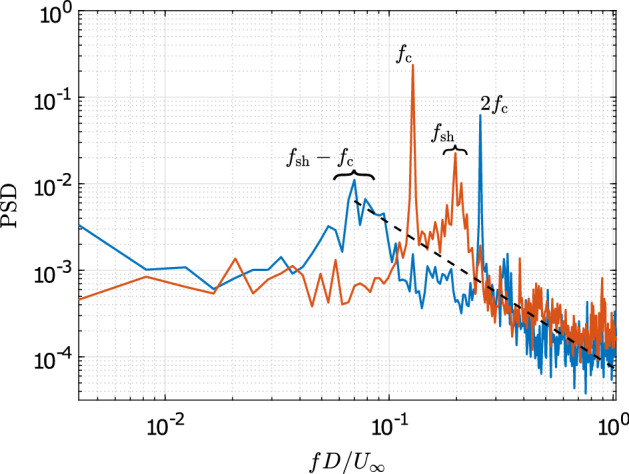
The power spectra (By Parseval’s identity, the integration of the power spectral density (PSD) over the frequency (in Hz) returns the variance of the signal.) of the measured velocity fluctuations at a point $x/D=1.19, y/D=-0.04$ (where $\overline{{u_i}'{u_i}'}$ is maximum): *u*-component in blue and *v*-component in orange. The dashed, black line shows the theoretical decay according to Kolmogorov’s -5/3 power law

A representative instant (PIV snapshot) of the wake flow is depicted in Fig. 4. Here, instantaneous streamlines overlay the iso-contours of the *v*-component of the velocity. The wake shows periodically shed vortices. The perturbations due to the cylinder displacement *s*(*t*) interact with these structures. These interactions result in an asymmetrical displacement of vortices. To describe the dynamics of the perturbed vortex street, several modes in combination are required. The observed asymmetry is expected to be captured by at least one mode, which needs to be considered in the mode selection process. The turbulent nature of the wake flow can be inferred from Fig. 5. Shown are the power spectra of both velocity components for a representative point ($x/D=1.19, y/D=-0.04$) where $\overline{{u_i}'{u_i}'}$ is maximum. The dominant frequencies in the *v*-component can be associated with the cylinder oscillation ($f_\mathrm {c}$) and the natural vortex shedding ($f_\mathrm {sh}$). The energy associated with the mechanical cylinder oscillations is concentrated in a narrow frequency band, while that for the vortex shedding exhibits the typical spectral broadening associated with perturbations due to a stochastic process. In the *u*-component, dominant frequencies are the second harmonic of the cylinder oscillation ($2f_\mathrm {c}$) and an inter-harmonic, $f_\mathrm {sh}-f_{c}$. The excitation of the inter-harmonic is associated with interaction of the cylinder motion and the vortex shedding. Approximately 40% of the spectral energy content is associated with the broad-band stochastic fluctuations. The main challenge in the form of the ROM is its ability to represent the interactions relating these dominant frequencies. In the following, the resulting model and flow representation are discussed in greater detail.

**Fig. 6:**
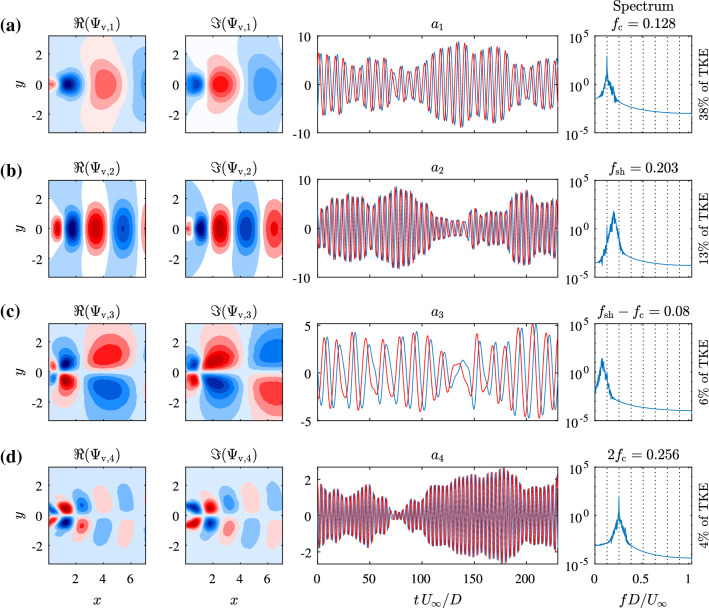
(left two columns) the spatial modes of the *v*-component of the four most energetic coupled SPOD modes of the velocity field, (mid) a representative time interval showing the evolution of the temporal coefficients $a_i$ and (right) power spectra of $\Re {(a_i)}$

### SPOD results

Figure 6 illustrates the SPOD modes, which consist of coupled mode pairs using DMD as described in Sect. 2.1. Shown are the *v*-component of the spatial (paired) mode, the evolution of the corresponding temporal coefficients and the spectrum of $\Re {(a_i)}$, which is representative for the coupled mode as the spectrum of $\Im {(a_i)}$ is almost identical. The first mode in Fig. 6 corresponds to the cylinder oscillation, as $f_\mathrm {c}$ matches the peak frequency of the independently measured lift force. The second mode is associated with the vortex shedding process, noting that its spectral energy is concentrated at the vortex shedding frequency $f_\mathrm {sh}$ and the distribution of extrema in its spatial component matches the number of vortices depicted in Fig. 4. Similar to the measure power spectrum in Fig. 5, the peak of the vortex shedding frequency shows a broader bandwidth. This indicates some sort of variation or disturbance in the vortex shedding process. Generally, the envelope of the oscillations of the first mode tends to be inversely related to the amplitude of oscillations of the second SPOD mode suggesting an energy exchange, or interference, between these modes. The frequency of the third mode corresponds to the difference of the vortex shedding and cylinder oscillation frequencies ($f_\mathrm {t}=f_\mathrm {sh}-f_\mathrm {c}$ from Fig. 6). Hence, the slow varying third mode is considered to represent an interaction between the first and second SPOD mode. This interaction is expected to increase the cycle-to-cycle variation in the vortex shedding frequency and lead to a broader frequency peak in the power spectra around the shedding frequency as can be observed in the measurement (Fig. 5). The spatial component of this mode is, in contrast to the previous two, asymmetrical. This mode can account for an asymmetry shift in the flow field observed in Fig. 4. The fourth SPOD mode corresponds to the second harmonic, $2f_\mathrm {c}$, of the cylinder oscillation. Its energetic content is relatively low and the amplitude of the oscillation, or envelope, appears directly related to that of the first mode. For the SPOD, a filter length $N_\mathrm {f}=32$ was used, which corresponds to two periods of the cylinder oscillation. With this choice, a good separation of the dynamics is already visible, as the mode spectra show very distinct and narrow peaks, while representing a significant amount of total fluctuation energy. Thus, the temporal coefficients are better behaved than the coefficients of a classical POD and therefore more suitable for the construction of a ROM. The influence of the SPOD filter lengths is discussed in “Appendix B”. Within the scope of illustrating the methodology, only the three most energetic coupled modes are selected to derive the ROM, as this is the minimal group of modes for a meaningful representation of the process and they already describe a complex interaction. As indicated with the fourth mode, additional coherent motions are captured in modes with significantly less energy content (low impact on the dominating dynamics), so that with the chosen degree of complexity these modes are not represented by the model.

### Final reduced-order model

The sparsification procedure starts with building a polynomial function library of cubic order, as illustrated in Eq. (27) using the three most energetic modes depicted in Fig. 6. The conservative and restrictive sparsification algorithms (Algorithms 1, 2) are applied to retain only the statistically significant library coefficients and corresponding functions. With this step, the number of possible library coefficients is reduced from 252 to only 15. In a final step, the model is calibrated against all available data. The resulting selection is presented in Table 1 and the corresponding system of ODEs in Eq. (38). Table 1 shows the coefficients, the calibrated values, the corresponding library functions and the system residual change $Q^{\text {sys}}$ of the coefficient. The system residual change represents the statistical relevance of an individual coefficient for the system description. The determination of $Q^{\text {sys}}$ follows the idea of the restrictive sparsification procedure, i. e. evaluating the relevance of the coefficients by comparing the model performance including and excluding the coefficient. For this, the measurement data are split into $N_\text {segs}$ equal-length segments for which the model is simulated. To avoid an over weighting of a specific cylinder oscillation phases, the initial values of the integration are shifted randomly within a period. The system residual change for each coefficient is determined as:

37 $$\begin{aligned} Q^{\text {sys}}=\frac{1}{N^\text {segs}}\sum _{n=1}^{N^\text {segs}}\frac{R^{\text {coeff}}_n-R^{\text {all}}_n}{R^{0}_n}, \end{aligned}$$

**Table 1:** Final selection of statistically significant library functions and corresponding library coefficients normalised with $D/(U_{\infty }2\pi )$, ordered by the system residual change $Q^{\text {sys}}$

No.	Mode	Coeff.	Library function	$Q^{\text {sys}}$ in %	Coeff. value
1.	$\dot{a}_{1}$	$\alpha _{1}$	$a_{1}$	46.565	$0.00518+0.13177i$
2.	$\dot{a}_{2}$	$\beta _{1}$	$a_{2}$	35.383	$0.00028+0.20098i$
3.	$\dot{a}_{3}$	$\gamma _{1}$	$a_{3}$	21.71	$-0.00450+0.07941i$
4.	$\dot{a}_{3}$	$\gamma _{2}$	$a_{2}a^{*}_{1}$	1.281	$0.00194+0.00021i$
5.	$\dot{a}_{3}$	$\gamma _{3}$	$a_{3}|a_{1}|^2$	1.259	$-0.00010+0.00030i$
6.	$\dot{a}_{2}$	$\beta _{2}$	$a_{2}a_{2}a^{*}_{1}$	1.053	$-0.00011-0.00036i$
7.	$\dot{a}_{1}$	$\alpha _{2}$	$a_{2}|a_{2}|^2$	0.99	$0.00003+0.00015i$
8.	$\dot{a}_{2}$	$\beta _{3}$	$a_{2}a_{3}$	0.793	$0.00247+0.00058i$
9.	$\dot{a}_{2}$	$\beta _{4}$	$a_{1}a_{1}a^{*}_{3}$	0.612	$-0.00003-0.00008i$
10.	$\dot{a}_{1}$	$\alpha _{3}$	$a_{1}|a_{1}|^2$	0.232	$-0.00005-0.00004i$
11.	$\dot{a}_{1}$	$\alpha _{4}$	$a_{1}|a_{2}|^2$	0.218	$-0.00015-0.00006i$
12.	$\dot{a}_{1}$	$\alpha _{5}$	$a_{2}a_{3}a^{*}_{1}$	0.095	$0.00002-0.00004i$
13.	$\dot{a}_{2}$	$\beta _{5}$	$a_{1}|a_{3}|^2$	0.076	$-0.00017+0.00026i$
14.	$\dot{a}_{3}$	$\gamma _{4}$	$|a_{1}|^2$	0.071	$0.00004+0.00005i$
15.	$\dot{a}_{1}$	$\alpha _{6}$	$a_{1}a_{1}a^{*}_{3}$	0.059	$0.00002+0.00004i$

where $R^{\text {all}}$ is the residual of the model using all selected coefficients, $R^{\text {coeff}}$ is the residual excluding the analysed coefficient and $R^{0}$ is the variance of the measured signal. With the coefficients in Table 1, the final system of ODEs then reads:

38a $$\begin{aligned} \frac{{\mathrm {d}} a_{1}}{{\mathrm {d}} t}&= \alpha _1 \cdot a_{1}+\alpha _2 \cdot a_{2}|a_{2}|^2+\alpha _3 \cdot a_{1}|a_{1}|^2+\alpha _4 \cdot a_{1}|a_{2}|^2+\alpha _5 \cdot a_{2}a_{3}a^{*}_{1}+\alpha _6 \cdot a_{1}a_{1}a^{*}_{3} \end{aligned}$$

38b $$\begin{aligned} \frac{{\mathrm {d}} a_{2}}{{\mathrm {d}} t}&= \beta _1 \cdot a_{2}+\beta _2 \cdot a_{2}a_{2}a^{*}_{1}+\beta _3 \cdot a_{2}a_{3}+\beta _4 \cdot a_{1}a_{1}a^{*}_{3}+\beta _5 \cdot a_{1}|a_{3}|^2 \end{aligned}$$

38c $$\begin{aligned} \frac{{\mathrm {d}} a_{3}}{{\mathrm {d}} t}&= \gamma _1 \cdot a_{3}+\gamma _2 \cdot a_{2}a^{*}_{1}+\gamma _3 \cdot a_{3}|a_{1}|^2+\gamma _4 \cdot |a_{1}|^2 \end{aligned}$$

The usage of specific library functions in combinations with the library coefficients provides insights into the flow physics. Here, the final selection (Table 1) can be categorised into different groups. The first and most important group with respect to the impact on the system are linear terms, which describe in this context a complex oscillator. The pairing of two real-valued coefficients to one complex-valued coefficient is introduced to link modes in the model, which are physically connected. On the level of the model ODE, this drastically simplifies how the oscillatory mode pair is represented. The description of linear oscillator by a pair of real-valued coefficients reads as follows [21, 24]:

39 $$\begin{aligned} \frac{{\mathrm {d}} }{{\mathrm {d}} t}\begin{bmatrix} a_{r} \\ a_{i} \\ \end{bmatrix}&= \begin{bmatrix} \sigma &{} -\omega \\ \omega &{} \sigma \\ \end{bmatrix} \begin{bmatrix} a_{r} \\ a_{i} \\ \end{bmatrix}, \end{aligned}$$

where $a_r$ and $a_i$ constitute the real and imaginary part of the coupled mode-pair $a = a_r + \mathrm {i} a_i$. The corresponding linear amplification rate $\sigma$ and oscillation frequency $\omega$ can be similarly composed to a complex coefficient $\alpha = \sigma + \mathrm {i} \omega$. This results in the simple representation of the complex oscillator as

40 $$\begin{aligned} \frac{{\mathrm {d}} a}{{\mathrm {d}} t} = \alpha a. \end{aligned}$$

Thus, for the linear terms, the imaginary part of the library coefficient determines the frequency of the corresponding mode. The frequencies of the modes derived by the calibrated library coefficients presented in Table 1 deviate less than 3% from the peak frequencies of the spectra in Fig. 6.

Similar to a nonlinear oscillating system described by the Stuart–Landau equation [16] and the generalised mean-field model [21, 24], the ROM includes nonlinear terms, where the mode $a_j$ is coupled with the amplitude but not the phase of a mode $a_k$:

41 $$\begin{aligned} \frac{{\mathrm {d}} a_j}{{\mathrm {d}} t} = +\cdots a_{j}|a_{k}|^2 +\cdots . \end{aligned}$$

Within the model, these terms address nonlinear coupling that results in changes of the linear terms described above. Thus, they influence the energy transfer between the modes, which can lead to a saturating effect stabilising the system. From a perspective of the flow dynamics, these terms can be understood as a mean field correction [24].

Further investigations of the coefficients show, for example for mode $a_3$ of Eq. (38c), a three mode interaction of the two complex coefficients $a_1 = A_1 \mathrm {e}^{\mathrm {i} \omega _1 t}$ and $a_2 = A_2 \mathrm {e}^{\mathrm {i} \omega _2 t}$, which results in the observed frequency mixing. This is clearly seen from the complex-valued multiplication

42 $$\begin{aligned} a_2a_1^* = A_2 \mathrm {e}^{\mathrm {i} \omega _2 t} A_1 \mathrm {e}^{-\mathrm {i} \omega _1 t} = A_1 A_2 \mathrm {e}^{\mathrm {i}(\omega _2-\omega _1)t}, \end{aligned}$$

represented by coefficient $\gamma _2$ of the model. It represents a forcing of mode $a_3$ at the difference of the individual frequencies, which corresponds exactly to the observed composition of mode frequencies in Fig. 6c, where the difference of forced shedding ($a_1$) at $f_\mathrm {c}=0.128$ and natural shedding ($a_2$) at $f_\mathrm {sh}=0.203$ leads to the interaction mode frequency of $f_\mathrm {t} \approx 0.203-0.128 \approx 0.08$. The modelling process identifies mode $a_3$ with a negative real part in the linear coefficient $\gamma _1$. Therefore, this interaction mode is actually damped and only observed due to the forcing by the interaction of cylinder oscillation $a_1$ and the natural vortex shedding $a_2$.

To provide another example for the interpretation of the model coefficients, $\beta _4$ ($a_1a_1a_3^*$) is discussed. It describes a triadic interaction of the second harmonic of the cylinder oscillation $a_1a_1$ with the interaction mode $a_3$ acting on the vortex shedding mode $a_2$. It corresponds to a frequency of $2\times 0.128-0.08 = 0.176$, which will be further discussed with the dynamics of the model presented in the following section. Note that the physics discussed only represents correlations in the data and most likely, but not necessarily, represents actual fluid dynamic interactions.

In a comparable way, other terms can be analysed and their contributions assessed, which helps to conceptualise internal mechanisms and interaction of the underlying physics.

### Model predictions

There are two aspects that need to be considered when assessing the performance of the ROM. First, the reduced number of modes used for the model only represents around ${60}{\%}$ of the turbulent kinetic energy (TKE) of the flow. Thus, only the most energetic and dominating flow structures are represented by the model and other coherent, and low-energy structures are not captured. Secondly, the model does not account for any random perturbation. Hence, the stochastic characteristics of the turbulence make an accurate long-term prediction with a deterministic model impossible. The deviations from constant amplitude (limit-cycle) oscillations are similarly observed for an isolated global instability in a turbulent flow [17, 36]. There, the deviations from the limit-cycle are explained by turbulent perturbations and related to the turbulence intensity.

**Fig. 7:**
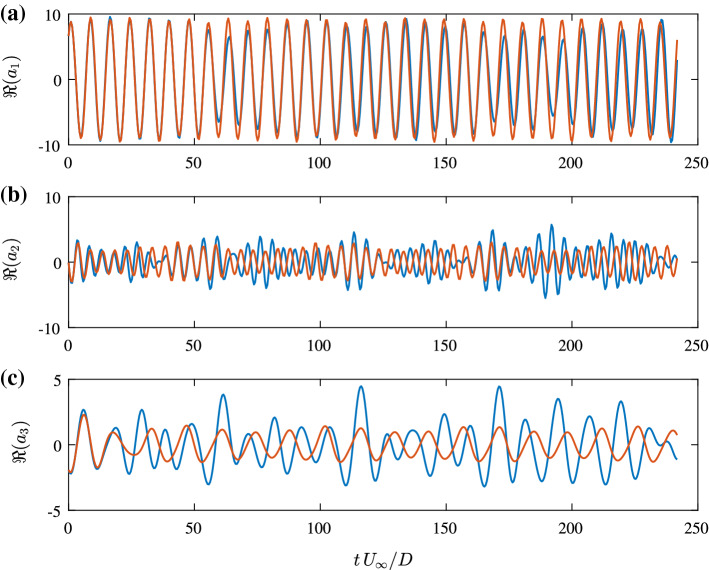
Representative time interval showing the temporal evolution of the real part $\Re (a_i)$ of the three most energetic SPOD modes (blue) and its prediction with the final ROM (orange); modes related to **a** cylinder oscillation, **b** the vortex shedding **c** their interaction

As a consequence, simulations of the final ROM often start to deviate from the actual temporal coefficients after a short period. In Fig. 7, observed and predicted evaluations of the real part of the temporal coefficients of the mode pairs are compared for representative time-series excerpts. Note, that the second part of the mode pair $\Im {(a_i)}$ shows the same behaviour. Generally, the higher-energy modes are better approximated over longer time intervals. For the lower-energy modes, for example $a_3$ in Fig. 7c, this deviation can be observed after two periods of the cylinder oscillation.

**Fig. 8:**
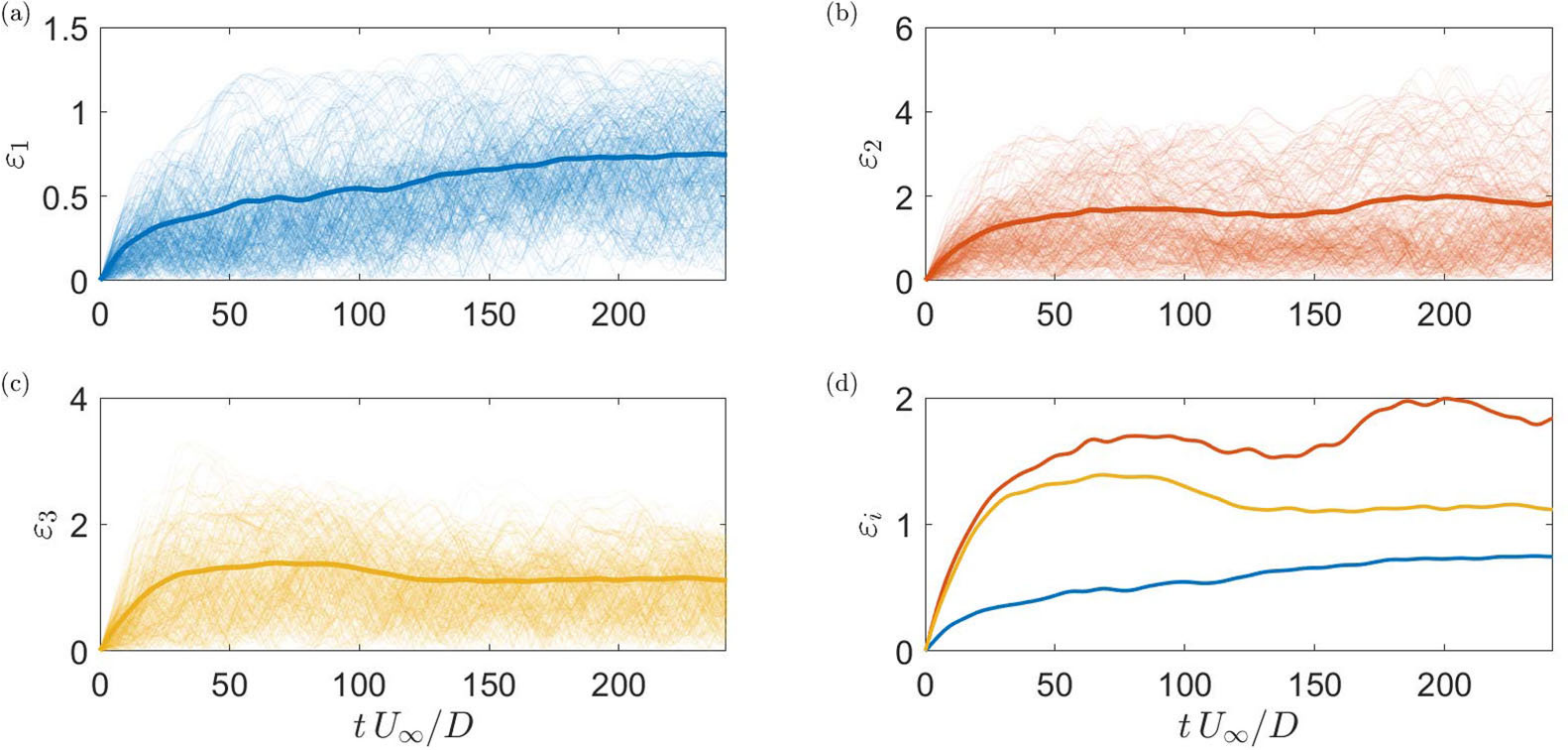
Residual of various simulations as thin lines (different initial values) and the averaged simulation residual (thick lines) of the calibrated ROM for the three most energetic SPOD modes; **a** the mode related to the cylinder oscillation (blue), **b** the vortex shedding mode (orange), **c** the interaction mode (yellow) and **d** the averaged simulation residual of all three modes

A more general statement of the performance can be obtained from the averaged simulation residual (for $N^\text {init}=300$ randomly chosen initial values):

43 $$\begin{aligned} \varepsilon _i =\frac{\sqrt{\frac{1}{N^\text{ init }}\sum _{n=1}^{N^\text{ init }}\left| a_i-a_i^\text{ sim }\right| ^2}}{\sqrt{\frac{1}{N^\text{ init }}\sum _{n=1}^{N^\text{ init }}\left| a_i\right| ^2}}, \end{aligned}$$

as displayed in Fig. 8. The gradient of the residual provides information on how fast the predictions deviate and to which extent the measurements are governed by stochastic influences. The convergence of the model to a certain error level indicates a general stability, as there is no unlimited growth independently of the initial values.

**Fig. 9:**
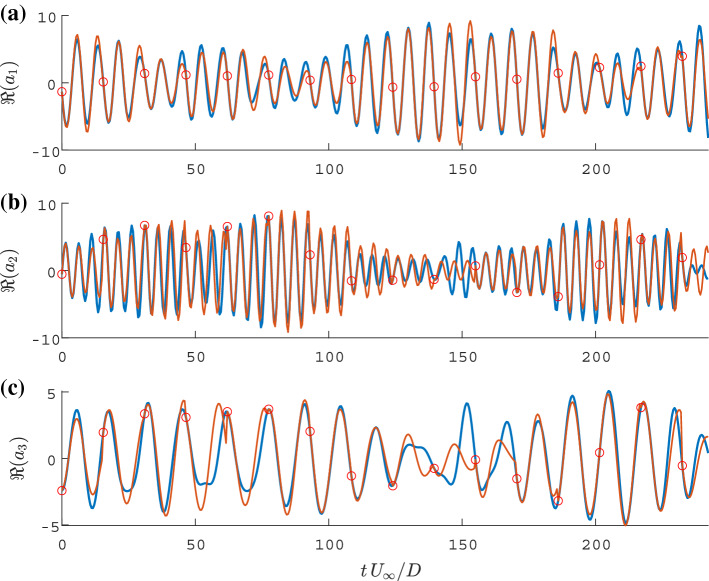
Representative time interval showing the temporal evolution of the real part $\Re (a_i)$ of the three most energetic SPOD modes (blue), a concatenated prediction $a_i^{\text {sim}}$ with the final ROM (orange) and the initial values used for the simulations (red circles); modes related to **a** cylinder oscillation, **b** the vortex shedding **c** their interaction

**Fig. 10:**
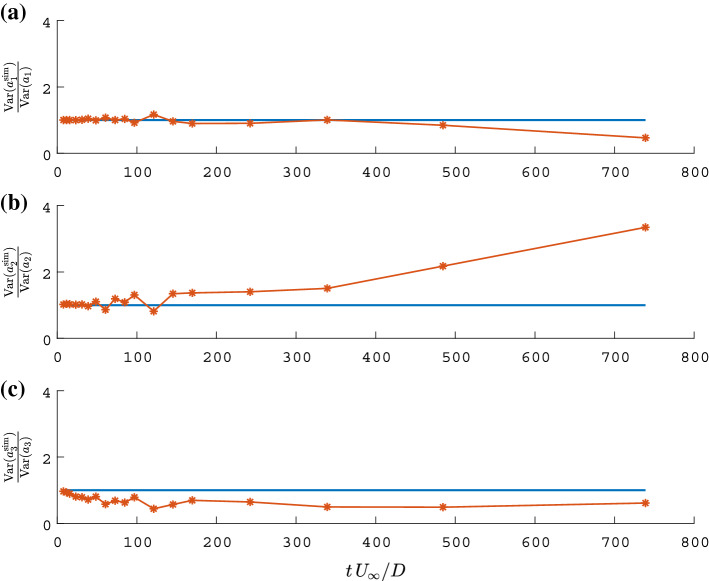
Variance of the original temporal coefficient (blue) and variance of reconstructed signals with different simulations lengths (orange, computed simulations lengths are indicated with a star); modes related to **a** cylinder oscillation, **b** the vortex shedding **c** their interaction

**Fig. 11:**
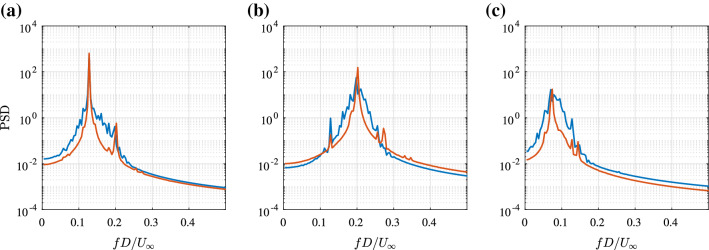
Power spectrum of the mode coefficient $\Re {(a_i)}$ obtained from the SPOD of PIV measurement (blue), a simulation of the calibrated low-order model (orange); modes related to **a** cylinder oscillation, **b** the vortex shedding **c** their interaction

The limitation of the long-term prediction accuracy raises the question: How precise can the statistics of the flow dynamics be represented by the model? To address this, simulations were conducted over equal-duration segments of the time series. The simulations were reinitialised at the beginning of each segment. The concatenation $a_i^{\text {sim}}$ of the simulated segments reconstructs the entire time series of the temporal coefficients. Figure 9 depicts samples of the observed coefficients overlaid with the concatenated segments. With a variation of the simulation duration, i. e. increasing the length and reducing the number of segments, one can evaluate how well the deterministic model performs compared to the measured statistics. Figure 10 displays the variance for each mode based on the measurement and the concatenated model prediction $a_i^{\text {sim}}$ corresponding to the representative example shown in Fig. 9. The definition of variance is given by:

44 $$\begin{aligned} \text {E}\left( \mathbf {X}\right)&=\frac{1}{N}\sum _{i=1}^{N}X_i =\mu , \end{aligned}$$

45 $$\begin{aligned} \text {Var}(\mathbf {X})&=\frac{1}{N-1}\sum _{i=1}^{N}|X_i-\mu |^2, \end{aligned}$$

where $\text {E}\left( \mathbf {X}\right)$ is the expected value of the signal and $\text {Var}\left( \mathbf {X}\right)$ its variance. It is a representation of the average mode energy. Hence, the deviation of the predicted from the actual (measured) variance provides insight of the model energy balance. For short simulations ($tU_\infty /D\le 50$), Fig. 10 shows a very good agreement of the actual and simulated variance. This is in line with the expectation, since for these time intervals the influence of stochastic perturbations is considerably lower. However, for longer simulations the prediction of the energy balance within the model differs from that of experimental data. Here, the downward drift of the first mode Fig. 10a and the upward drift of the second mode Fig. 10b indicate that in the model more energy is transferred from the first to the second mode than observed in the data. For each segment of the concatenated coefficients, i.e. for each simulation of the temporal coefficient, a fast Fourier transform (FFT) of $\Re ({a_i})$ is computed. The average of these spectra (for $tU_{\infty }/D \approx 240$) is shown in Fig. 11. These spectra are representative for the mode pair and correspond to relatively long simulations, i. e. long segments (30% of the realisation data) and show that the model still captures the general, spectral characteristics of the flow. The difference between the predicted and the measured spectra can be attributed to several factors. The deviation is most obvious in the decay of the flanks next to the peak. The model only explains a narrow spectral contribution at the peak frequency of each mode, whereas the observed (measured) mode coefficients include further dynamics. The previously illustrated triadic interaction represented by the coefficient $\beta _4$ of the model describes a forcing of the vortex shedding mode at a frequency of 0.176. This causes the hump to the left of the main peak of the simulated spectrum in Fig. 11b. However, such nonlinear mechanisms explain only part of the measured spectral broadening. Since the model only includes the first three SPOD mode pairs, which account for about 60% of the turbulent kinetic energy, the difference could be due to the neglected effect of higher modes on the leading modes. The model thus captures the interactions between the large amplitude motions, but does not explicitly account for lower energy perturbations. The discarded modes, representing further coherent dynamics or stochastic fluctuations, might thus be necessary to model the observed broader spectral peaks. Instead of adding further modes, the unresolved perturbations can be modelled by a generic stochastic forcing, which effectively emulates the observed broadening of the spectral peaks [25]. This approach does not improve the predictive capabilities of the model since it only adds random perturbations. However, it allows better reproduction of the average statistics [36]. In this context, it should be noted that, although the perturbations can be considered as additive random forcing for modelling purposes, in the experimental data they are not filtered out by the decomposition. The data were recorded from the entire system including the disturbances. Therefore, the unresolved disturbances are implicitly included as frequency fluctuations of the resolved data.

While there could also be a broadening of the spectra due to measurement noise, this effect is expected to be secondary, as such contributions are generally poorly correlated in space and time and are filtered out by the modal decomposition [32]. Regardless of the reason for the discrepancy, obtaining directly a more accurate model would be at the cost of a significant increase in its complexity. The potential benefit of using just a few additional modes is usually small. Since the remaining modes contain much less energy, substantial differences in the model usually require many more modes. Furthermore, this mainly translates the problem of the neglected action of unresolved modes on resolved modes towards higher modes. The alternative representation by generic stochastic forcing requires further modelling of the forcing characteristics, which is not straightforward [2, 6]. The choice of the right model complexity ultimately depends on the intended purpose of the ROM, balancing captured dynamics and predictive accuracy against complexity.

**Fig. 12:**
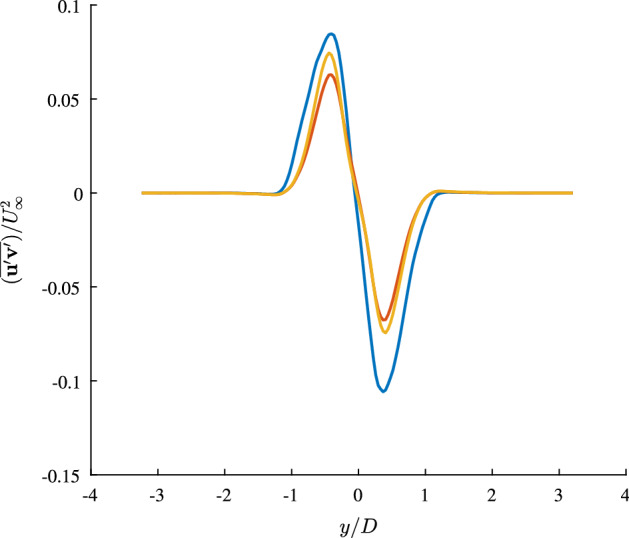
Cross-section of the maximal shear Reynolds stresses at $x/D=0.63$ obtained from the PIV measurement (blue), and the calibrated ROM using the first three SPOD modes (orange) and a piece-wise reconstructed flow field with simulations of 500 time steps (yellow)

With the simulated temporal coefficient and the corresponding spatial modes, a simulated velocity field can be reconstructed using Eq. (21). This allows to estimate the Reynolds stresses in the cylinder wake and gain some insight of predicted flow physics. Figure 12 displays the shear Reynolds stresses at the cross section with the maximum measured Reynolds stresses for: the measured field, a reconstructed field using the first three SPOD modes and the stresses reconstructed with the simulated temporal coefficients. The estimation of the shear stresses provides a measure to evaluate if the model can represent the influence of the large flow structures. Using the measured temporal coefficients to reconstruct the flow field provides a reference for comparison.

As both reconstructed Reynolds stresses account only for around $\sim {60}{\%}$ of the TKE, the extrema of these stresses are expected to be lower than the stresses derived from the measurement. However, the simulated stresses are already very close to the stresses described with the three SPOD modes. Slightly reducing the simulation length leads to an excellent match of the reconstructed stresses. Thus, with respect to the represented energy content, the deterministic ROM is capable of accurately reproducing the statistics associated with the flow physics. Further, for this flow configuration the influence of the stochastic perturbations is limited to the extend that the dominating dynamics can be captured with a few modes and represented by the model.

## Concluding remarks

A robust method has been presented for deriving reduced order models from data to describe the underlying flow dynamics. The methodology is demonstrated for the turbulent wake of a cylinder undergoing vortex-induced vibration. Its robustness is achieved by establishing objective criteria in the decision process underpinning a two-step sparsification procedure. This approach aims to minimise user interaction and ensure an unambiguous procedure.

The separation of the flow dynamics is realised by a modal decomposition of the flow data. A reduced set of spatial modes represents dominating flow structures and their dynamics through their corresponding temporal coefficients. The evolution of these coefficients allows a simplified description of the dynamics. A system of ODEs is constructed using linear and nonlinear combination (here polynomial) of the coefficients to model the dynamics. These combinations define a library of possible functions. The critical step in the presented method is the optimised selection (sparsification) process to identify the minimal number of relevant library functions and corresponding coefficients implementing a two-stage cross-validation procedure. The first stage (conservative sparsification) works as a prefiltering of the library coefficients, where all of these coefficients are step-wise tested and relevant coefficients with respect to the approximation of the derivative are kept. In the second stage (restrictive sparsification), an iterative process is used to quantify the relevance of each library coefficient for the system and remove non-physical coefficients. Briefly, these coefficients are used to build models, validated them against the measured evolution of the temporal coefficients and perform short-time predictions. The final selection of library coefficients follows a statistical significance test by the successive exclusion of coefficients.

The major improvement gained through the sparsification procedure addresses three challenges in particular. Firstly, in contrast to previous approaches, it not only identifies coefficients that influence the approximation of the derivative, but also determines how sensitive the prediction is to a given coefficient. This enhances the reliability and robustness of the final ROM. Secondly, introducing cross-validation in both stages of the sparsification reduces the risk of overfitting. Within the conservative sparsification, this is accomplished by using randomly chosen segments of the computed derivative to train the coefficients and the non-training segments for validation. For the second stage, the training is performed in a similar way, but the validation is based on the prediction of the temporal evolution of the non-training segments and not its derivative. Using only 20% of the data for training and 80% for the validation further minimises the risk of overfitting. Finally, a central goal of the new sparsification algorithms is the definition of an objective criterion for the termination of the sparsification procedure. Thus, the system identified is not sensitive to user-selected thresholds and the complexity of the model is determined by the data.

The choice of decomposition technique is not considered essential to the process. However, the proprieties of the decomposition do influence the general performance of the ROM. Here, SPOD was chosen because it enables an efficient separation of the dynamics and leads to well-behaved temporal coefficients. This is beneficial for the construction of ROM and increases the probability, that the process results in an accurate and robust representation of dominating flow dynamics. Another optional step is the identification and coupling of modes pairs with a DMD and their representation as a complex mode. By minimising the number of possible library functions and forcing the process to handle modes as paired couples, the computational costs in the sparsification procedure are reduced and the description of the system simplified. The procedure can be applied using real valued modes and without prior coupling.

The number of selected modes is a balance between model complexity and the dynamics resolved by the model. Note, this applies to all approaches based on modal decomposition. Additional models using a larger number of modes have also been computed. The resulting models are more complex but benefit the representation by incorporating interactions between the higher energy modes and the added modes. However, it remains that the more complex models do not change the basic form and relationships between the modes presented in this work. Consequently, the simpler model is found to be more suitable for an initial demonstration of the method.

The results from the presented method show its capability to derive a robust ROM solely from measurement data. It can capture the deterministic part of the underlying dynamics of a turbulent wake. The only exogenous parameters that need to be defined within the process can either be derived from dynamical characteristics (e.g. vortex shedding frequency) of the flow or address procedural adjustments such as the number of repeated iterations on different parts of the data. Moreover, the process design exhibits a low sensitivity to control parameter variations. Hence, the process converges towards a final model, i. e. robustly identifying the same basis functions and library coefficients repeatably. Additionally, the analytic structure of the utilised library functions resembles the terms of a Galerkin projection of the Navier–Stokes equations. Thus, it enables to gain insight of the model mechanics and interactions by analogy. Since specific terms of the ROM can be related to fluid mechanical phenomena, the model also allows an interpretation of flow physics. Due to its fast prediction capability, the model may potentially be suitable for applications where rapid approximations of the flow evolution are critical, such as for active flow control, process monitoring and assessing design performance

## Figures and Tables

**Fig. 13:**
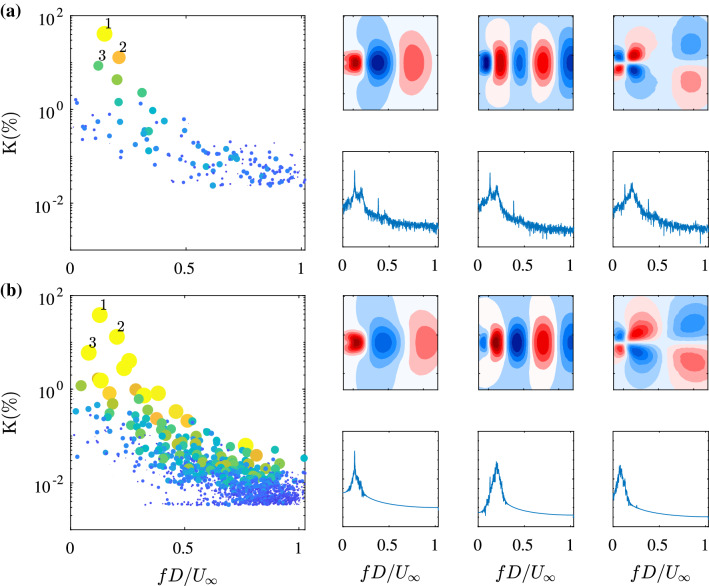
Resulting SPOD spectrum for different filter length: **a** $N_\mathrm {f}=0$ (POD) and **b** $N_\mathrm {f}=32$ (SPOD) displayed as a scatter plot, where a single dot indicates one couple SPOD mode pair and the size and colour refer to the harmonic correlation Eq. (18) of the modes. For both filter lengths, three mode pairs are selected (indicated by numbers in the SPOD spectrum) and their spatial mode of the *v*-component (upper row) and the PSD of the corresponding temporal $\Re (a_i)$ (lower row)

## A Symmetrisation

In case the investigated flow field shows a symmetry, it is recommended to make use of symmetrical properties. This enables an improved separation of the dynamics with a modal decomposition. The wake of a circular cylinder undergoing VIV showed a symmetry with respect to the $y=0$-plane. Thus, before performing a modal decomposition, the fluctuation is decomposed into a symmetric $\mathbf {v}'^{(s)}(\mathbf {x},t)$ and an asymmetric $\mathbf {v}'^{(a)}(\mathbf {x},t)$ part.

46 $$\begin{aligned} \mathbf {v}(\mathbf {x},t)&=\overline{\mathbf {v}(\mathbf {x})}+\mathbf {v}'(\mathbf {x},t) \end{aligned}$$

47 $$\begin{aligned} \mathbf {v}'(\mathbf {x},t)&=\mathbf {v}'^{(s)}(\mathbf {x},t)+\mathbf {v}'^{(a)}(\mathbf {x},t) \end{aligned}$$

The symmetric and asymmetric split is computed as follows:

48a $$\begin{aligned} u'^{(s)}(x,y,z,t)&=\frac{1}{2}\left[ u'(x,y,z,t)+u'(x,-y,z,t)\right] \end{aligned}$$

48b $$\begin{aligned} u'^{(a)}(x,y,z,t)&=\frac{1}{2}\left[ u'(x,y,z,t)-u'(x,-y,z,t)\right] \end{aligned}$$

48c $$\begin{aligned} v'^{(s)}(x,y,z,t)&=\frac{1}{2}\left[ v'(x,y,z,t)-v'(x,-y,z,t)\right] \end{aligned}$$

48d $$\begin{aligned} v'^{(a)}(x,y,z,t)&=\frac{1}{2}\left[ v'(x,y,z,t)+v'(x,-y,z,t)\right] \end{aligned}$$

48e $$\begin{aligned} w'^{(s)}(x,y,z,t)&=\frac{1}{2}\left[ w'(x,y,z,t)+w'(x,-y,z,t)\right] \end{aligned}$$

48f $$\begin{aligned} w'^{(a)}(x,y,z,t)&=\frac{1}{2}\left[ w'(x,y,z,t)-w'(x,-y,z,t)\right] \end{aligned}$$

With both, POD and SPOD, the symmetric and asymmetric fluctuations can be described by a sum of spatial modes $\mathbf {\Phi }_i(\mathbf {x})$ and corresponding temporal coefficients $b_i(t)$: With Eqs. (46), ( 47) and ( 49), the symmetric and asymmetric part can be written in a combined sum:

49 $$\begin{aligned} \mathbf {v}'(\mathbf {x},t)&=\sum _{i=1}^{N}{\mathbf {\Phi }^{(s)}_{i}(\mathbf {x})b^{(s)}_{i}(t)}+\sum _{i=1}^{N}{\mathbf {\Phi }^{(a)}_{i}(\mathbf {x})b^{(a)}_{i}(t)}. \end{aligned}$$

Then, Eq. (49) can be written as a combined sum:

50 $$\begin{aligned} \mathbf {v}'(\mathbf {x},t)&=\sum _{i=1}^{2N}{\mathbf {\Phi }_{i}(\mathbf {x})b_{i}(t)}, \end{aligned}$$

where the modes are ordered by energy. From here on, the procedure continues as described in Sect. 2.

## B SPOD filter

SPOD results of the symmetrised VIV data for two filter lengths ($N_\mathrm {f}=0, 32$) are illustrated in Fig. 13. A limiting case, computed with the minimal filter length ($N_\mathrm {f}=0$), is equivalent to results of a classical POD. The case using $N_\mathrm {f}=32$ corresponds to the results discussed in Sect. 3. This filter length approximately corresponds to a characteristic time scale of the flow (two periods of the cylinder oscillation, $N_\mathrm {f}\cdot \varDelta t = {1.33}{s}, T_\mathrm {p} =1/f_\mathrm {c} \approx {0.67}{s}$). For each case, so-called SPOD spectra [35] are depicted in Fig. 13 (left side), where a single dot represents a coupled mode pair. The size of the dote scales with the magnitude of its harmonic correlation $H_{ij}$ determined according to Eq. (18). The colour also indicates the magnitude and is used to enhance visibility. The frequency *f* of the identified mode pair is computed by the weighted sum of the DMD eigenvalues $\nu _k$ (Eq. 14) sorted in descending order [35]:

51 $$\begin{aligned} f&=\frac{\sum \nolimits ^n_{i=1}\Im (\ln {\nu _i})\widetilde{h}_i}{2\pi \varDelta t \sum \nolimits ^n_{i=1}\widetilde{h}_i} \end{aligned}$$

52 $$\begin{aligned} \widetilde{h}_i&=h^2_{ij}+h^2_{ik} \end{aligned}$$

Here, an averaged over three eigenvalues ($n=3$) is used, to mitigate possible corruption by noise. The relative energy content *K*, with respect to the total energy, of the mode pair is given by:

53 $$\begin{aligned} K&=\frac{\mu _i+\mu _j}{\sum \nolimits ^{N_\mathrm {c}}_{k=1}\mu _k}. \end{aligned}$$

The subscripts *i* and *j* refer to the indices of the coupled SPOD modes. Three modes are selected (indicated by numbers), which are related to the cylinder oscillation (1), the vortex shedding (2) and their interaction (3). In Sect. 3.3, these modes are discussed in detail for $N_\mathrm {f}=32$. To the right of each SPOD spectrum (Fig. 13), the spatial mode shapes $\Phi _{v,i}(\mathbf{x} )$ (upper row) and the power spectral density (PSD) function of the real part of the corresponding temporal coefficients (lower row) are displayed for the three selected modes. The spatial mode shapes are visualised by the *v*-component of the velocity.

For a compact representation, the mode spectra and spatial mode shapes are given without axis labelling. The axis corresponds to analogous plots in Fig. 6.

The POD ($N_\mathrm {f}=0$) spectrum in Fig. 13a shows few modes with a high harmonic correlation at lower Strouhal numbers $St=fD/U\infty$ and a high energy content *K*. In the following, the effect of the filter size is primarily demonstrated for the selected modes. The spatial shapes of these modes are clearly symmetric (1) and (2) or anti-symmetric (3). The mode spectra of the corresponding temporal coefficients show broad bandwidth with several higher peak frequencies at different Strouhal numbers. Some of these frequencies are present in multiple spectra. For example, all three of the mode spectra show peaks at $St=0.128$ and $St=0.209$. This indicates that the dynamics are not fully separated.

In contrast, the SPOD ($N_\mathrm {f}=32$) spectrum yields similar modes with respect to the spatial mode shape, the peak frequency and energy content, but with a higher harmonic correlation, Fig. 13b. Only minor differences in the spatial shapes for the selected modes are observable, e. g. slight shifts in the distribution of the extrema. Note, that due to the previously applied symmetrisation, the effect of the filter operation on the spatial mode shapes is reduced. The mode spectra, however, are significantly changed. Instead of showing multiple high spectral peaks, Fig. 13a, each of the SPOD mode spectra is centred around a single frequency (Fig. 13b) with narrow bandwidth, so that the peaks, such as $St=0.128$ and $St=0.209$, are now associated with a specific SPOD mode. Briefly, when comparing SPOD to the POD modes, the spatial mode shapes and the energy content remain almost unchanged. However, the mode spectra indicate that with the SPOD a better separation of the dynamics can be achieved resulting in temporal coefficients with an increased harmonic correlation.

The filter size $N_\mathrm {f}$ is an important parameter of the SPOD. Gradually increasing $N_\mathrm {f}$ allows a continuous transition from POD to DFT. Accordingly, the spectral separation appears to be continuous, too. Selecting the filter size is a compromise between two competing effects. Increasing $N_\mathrm {f}$ increases the spectral separation at the cost of reducing the energy content of each mode. Thus, the selection of the filter size is not critical in the sense of finding a single optimal parameter, but identifying the range, where the dynamics are separated and the modes represent the most energy. Selecting the filter size based on a characteristic time scales of the flow showed suitable results for different flow configurations [35].

## C Numerical derivative

Assuming non-periodic boundary conditions, the coefficients for the sixth-order compact scheme at the boundary are defined as follows:

54 $$\begin{aligned} \begin{bmatrix} 1 &{} 5 &{} 0 &{} 0 &{}0 &{}\cdots \\ \frac{2}{11} &{} 1 &{}\frac{2}{11} &{} 0 &{}0 &{}\cdots \\ 0 &{}\frac{1}{3} &{} 1 &{}\frac{1}{3} &{} 0 &{}\cdots \\ &{} &{} \ddots &{}\ddots &{}\ddots &{} &{} \\ &{}&{} &{} &{} \ddots &{} \ddots &{}\ddots &{} \\ &{}&{} &{} &{} \cdots &{}\frac{2}{11} &{}1 &{}\frac{2}{11} \\ &{} &{} &{} &{} \cdots &{} 0 &{}5 &{}1\\ \end{bmatrix} \begin{bmatrix} \dot{a}_1\\ \dot{a}_2\\ \dot{a}_3\\ \vdots \\ \dot{a}_{N-2}\\ \dot{a}_{N-1} \\ \dot{a}_{N}\\ \end{bmatrix}= \frac{1}{\varDelta t} \begin{bmatrix} -\frac{197}{60} &{}-\frac{5}{12} &{} 5 &{}-\frac{5}{3} &{}\frac{5}{12} &{}-\frac{1}{20} &{}0 &{}\cdots \\ -\frac{20}{33} &{}-\frac{35}{132}&{} \frac{34}{33} &{}-\frac{7}{33} &{}\frac{2}{33} &{}-\frac{1}{132} &{}0 &{}\cdots \\ -\frac{1}{36} &{}-\frac{14}{18} &{} 0 &{}\frac{14}{18} &{} \frac{1}{36} &{} 0 &{}0 &{}\cdots \\ &{} \ddots &{} \ddots &{}\ddots &{}\ddots &{} \ddots &{} &{} &{} &{} \\ &{} &{} \ddots &{} \ddots &{} \ddots &{}\ddots &{}\ddots &{} \\ &{} \cdots &{} \frac{1}{132} &{}-\frac{2}{33} &{}\frac{7}{33} &{}-\frac{34}{33} &{}\frac{35}{132} &{}\frac{20}{33} \\ &{} \cdots &{} \frac{1}{20} &{} -\frac{5}{12} &{} \frac{5}{3} &{}-5 &{}\frac{5}{12} &{}\frac{197}{60} \\ \end{bmatrix} \begin{bmatrix} {a}_1\\ {a}_2\\ {a}_3\\ \vdots \\ {a}_{N-2}\\ {a}_{N-1} \\ {a}_{N}\\ \end{bmatrix} \end{aligned}$$

## D Experimental data

Velocity and force data were obtained from a study conducted at the University of Calgary [27]. A cyber-physical apparatus [8, 11, 44], described in Riches et al. [26], was used to operate a real-time simulation of the mechanical system described in Eq. (55)

55 $$\begin{aligned} m\ddot{y}+c\dot{y}+ky=F_y, \end{aligned}$$

where *m*, *c*, and *k* are the structural mass, damping, and stiffness, respectively. $F_y$ is a time-dependent forcing from the wake dynamics in the transverse direction of the incoming free stream flow [13]. The measured forces are used as an input to calculate the kinematics, so that the structural mass, stiffness and damping can be modelled in software. With a motorised traverse, the cylinder is then forced to follow these kinematics. The experiment was conducted to analyse the wake of a hollow acrylic cylinder (diameter $D={19.05}$ mm and length $L\approx 20D$) with a non-dimensionalised mass ratio $m^*$ and a damping ratio $\xi$:

56 $$\begin{aligned} m^*&=\frac{4m}{\pi \rho D^2L}=8 \end{aligned}$$

57 $$\begin{aligned} \xi&=\frac{c}{2\sqrt{km}}=0.005 \end{aligned}$$

at $\mathrm{Re}=4000$ and $U^*=8.75$. Data of the time-resolved two-component planar PIV were collected in a plane of the cylinder midspan. The images were captured with a high-speed Phantom Miro M340 digital camera ($2560 \times 1600$). The flow was seeded with 11.7$\mu {\mathrm{m}}$ hollow-glass spheres, and the plane was illuminated by a laser sheet from a Photonics 20 mJ Nd:YLF high repetition rate pulsed laser with a wavelength of $\lambda = {527}$ nm. Both, camera and laser were configured to operate in dual-pulse mode at an effective data image per acquisition rate of 24Hz. For the processing of the PIV images, the *LaVision DaVis* 8.3 software suite was used. The time-resolved velocity vector fields were calculated using a multi-pass cross-correlation algorithm with a final interrogation window size of $24 \times 24$ pixels resulting in a vector spacing of 0.725 mm. More information and details of experimental setup and technical components are available in Riches et al. [26] and [27]. Within three realisations of the experiment, a total number of 4767 vector fields (1589 per realisation) were obtained at a sample frequency of 24Hz comprising approximately 298 periods of the cylinder oscillation.
